# Prospective memory in Alzheimer’s disease and Mild Cognitive Impairment

**DOI:** 10.1590/S1980-57642011DN05020002

**Published:** 2011

**Authors:** Lívia Spíndola, Sonia Maria Dozzi Brucki

**Affiliations:** 1 Neuropsychologist, Post-Graduate Student. Department of Neurology, University of São Paulo School Medicine, São Paulo SP, Brazil.; 2 MD, PhD, Behavioral and Cognitive Neurology Unit, Department of Neurology, University of São Paulo School Medicine, São Paulo SP, Brazil.

**Keywords:** prospective memory, memory for intentions, dementia, Alzheimer’s disease, mild cognitive impairment

## Abstract

Prospective memory (PM) is defined as remembering to carry out intended actions at an appropriate point in the future, and can be categorized into three types of situation: time-, event-, and activity-based tasks. PM involves brain structures such as frontal and medial temporal cortices. The aim of this study was to review the currently available literature on PM in Alzheimer’s disease and Mild Cognitive Impairment patients. We performed a search on Pubmed, Medline, ScieLO, LILACS and the Cochrane Library electronic databases from January 1990 to December 2010. The key terms used were: prospective memory, memory for intentions, delayed memory and memory for future actions, separately and also combined with the search terms dementia, Alzheimer’s disease and Mild Cognitive Impairment. Both patient groups showed significant impairment in PM. Further studies are needed to verify the accuracy of PM tasks as an early marker of mild cognitive impairment, and initial dementia.

## Abstract (pt)

Memória prospectiva (MP) é lembrar-se de completar ações intencionadas num ponto do futuro, pode ser categorizada em três tipos de situação: tarefas baseadas em tempo, evento e atividade. MP envolve estruturas cerebrais como córtices frontais e temporais mediais. Este artigo teve como objetivo revisar a literatura disponível de MP na doença de Alzheimer e Comprometimento Cognitivo Leve. Nós realizamos uma pesquisa nas bases de dados: Pubmed, Medline, ScieLO, LILACS e Cochrane Library electronic de janeiro de 1990 a dezembro de 2010. Os termos chave usados foram: *prospective memory, memory for intentions, delayed memory e memory for future actions*, combinados com os termos *dementia, Alzheimer’s disease e Mild Cognitive Impairment*. Os grupos de pacientes demonstraram comprometimento importante em tarefas de MP. Mais estudos são necessários para verificar a acurácia de tarefas de MP como um marcador precoce de comprometimento cognitivo leve e demência inicial.

Prospective memory (PM) denotes the ability to remember and perform an intended action at an appropriate point in the future.^1^ Remembering to take medication, to go to the doctor for an appointment or to switch off the stove after cooking are examples of prospective memory. PM contrasts with retrospective memory, the ability to recall information or events from the past, such as recalling items of a grocery-shopping list.

Most studies that have examined the nature and extent of memory impairment in Alzheimer’s disease patients (AD) and other forms of dementia have focused on retrospective memory. However, PM has not been systematically evaluated.

PM is commonly engaged in everyday life and is highly relevant for maintaining functional independence, especially in older adults.^2^ Failure to perform future actions can have serious health consequences and negatively impact instrumental activities of daily living. Moreover, caregivers of individuals with Alzheimer’s disease report PM failures as being more frequent and more burdensome than retrospective memory failures.^3^

The variety of PM situations can be categorized into three types according to whether the cue for initiating the action is an event, a time or an activity.^4,5^

For event-based tasks, the intended action is performed when some external event occurs, such as remembering to give a friend a message when seeing them. Generally, in laboratory settings, subjects are asked to press a button when a target word or category appears, or after any of more than one (four, for example) target word appears.

For time-based tasks, the intended action is carried out at a specific time or after a set amount of time has elapsed, such as attending a doctor’s appointment at 10 a.m. or returning a phone call in 30 minutes. In laboratory time-based tasks, subjects are given an ongoing activity to perform. A possible task is pressing a key on the keyboard after a predefined amount of time. In real life scenarios, a similar situation would be to remember to turn off the burner after a 10-minute cooking time.

For activity-based tasks, the intended action is performed after the completion of another activity, such as remembering to take a pill after dinner; or to buy bread on your way home after work. An example experimental situation is to solve an arithmetic problem after writing two sentences.

Time-based PM has been consistently reported to be impaired in older adults. Einstein et al.^6^ suggested that this pattern may be attributable to the greater demands for self-initiated processing involved in time-based tasks, which lack any external reminders. This suggestion is in line with Craik’s^7^ proposal that self-initiated processing declines with age as a consequence of a decline in attentional resources, or possibly reflects an age-related decline in frontally-mediated cognitive control.^8^

McDaniel and Einstein^9^ suggested that successful prospective remembering requires two distinct components: a more purely prospective component, which entails remembering that something has to be done, and a second, more typically retrospective component, which involves remembering the content of an action. Whereas attention and executive processes are more involved in the prospective component of a task, the retrospective component of a task is mediated by retrospective memory abilities.

PM is a complex process involving four distinct phases: formation and encoding of an intention; intention retention (period during which the intention is retained in memory); initiating the intention; and the intention execution.^10^ Ellis^11^ proposed a fifth phase that is concerned with monitoring the output of an executed intention. Specific cognitive resources have been identified in each phase: planning, storage, monitoring, cognitive flexibility or inhibition. Craik suggested that PM tasks are the highest self-initiated retrieval demands because of an absence of cues, and a need to remember to remember.^7^ It involves cognitive processes that are mediated by prefrontal systems and are executive in nature.^10^

Indeed, neuropsychology studies suggest that PM performance depends on the functional integrity of the prefrontal areas and the effectiveness of executive functions.^12^ Disruption of the medial temporal lobe memory structures may also cause impairments in PM.^13^

These findings converge with recent functional neuroimaging studies demonstrating that PM processes are supported by a broadly distributed neural network involving structures within the rostral prefrontal cortex (particularly Brodmann’s area 10), the parietal cortex and the hippocampal complex.^14-16^

These areas are known to be affected in AD patients both structurally and functionally. The neuropathology of Alzheimer’s disease is characterized by the presence of amyloid plaques and neurofibrillary tangles.^17^ A recent study reported that this pathology is initially concentrated in medial prefrontal and parietal cortices, as well as lateral parietal and temporal regions.^18^

By definition, retrospective memory impairment is considered the most prominent symptom for AD.^19^ However, there is growing evidence that alterations in other cognitive functions such as executive function are also common and involve impairments in working memory and initiation, perseveration, problem solving, strategic planning and poor conceptual abilities.^20^

On the basis of these cognitive and pathological alterations, AD patients can be expected to exhibit substantial difficulties performing PM tasks.

The aims of this paper were to conduct a review of the currently available literature assessing PM in Alzheimer’s disease and Mild Cognitive Impairment (MCI) populations. A search on Pubmed, Medline, ScieLO, LILACS and the Co­chrane Library electronic databases spanning publications from January 1990 to December 2010, was performed. The key terms used were: prospective memory, memory for intentions, delayed memory and memory for future actions, separately and also combined with search terms dementia, Alzheimer’s disease and Mild Cognitive Impairment. The bibliographies of relevant papers were also examined for additional references.

## Prospective memory in Alzheimer’s disease

The first investigation of PM in patients with dementia was carried out by Huppert and Beardsall.^21^ These authors tested patients with minimal dementia, mild/moderate dementia and normal controls, on three measures of PM which form part of the Rivermead Behavioral Memory Test (RBMT; Wilson, Cockburn & Baddeley, 1985) as well as by retrospective memory tests. The cited study revealed that in retrospective memory, patients with minimal dementia attained intermediate scores compared to the other two groups. In contrast, on the prospective memory tasks, patients with minimal dementia showed a similar performance to that of moderately demented patients, with both patients groups scoring substantially worse than controls. Huppert and Beardsall concluded that PM tasks are particularly sensitive for the early stages of dementia, perhaps to a greater extent than are standard retrospective memory assessments.

In another study, Huppert et al.^22^ examined the prevalence of PM impairments in the elderly population. These authors administered three retrospective memory tests, and a single event-based PM task, to 11,956 older adults. The task required participants to carry out two actions, sealing and initialing an envelope when the envelope was re-presented during cognitive assessment. Only 54% of this sample succeeded on the task. The study found a high prevalence of PM impairment in 388 individuals with very mild dementia, of whom only 8% succeeded on the task.

Maylor et al.^23^ in an experimental study, compared PM and retrospective memory performance of AD patients, young and old controls. They found significant deficits for both event-based and time-based PM tasks in AD patients compared with age-matched controls. However, retrospective memory tasks (digit span, sentence span, free recall, and recognition) were more impaired in AD than were the PM tasks. The authors concluded there was no evidence that PM is especially impaired in AD compared with retrospective memory. However, AD patients in their study were not all in the early stages of dementia.

Some studies have investigated the relationship between presence of the ε4 allele of APOE (known genetic risk factor for AD) and PM. Driscoll et al.^24^ reported greater impairment on event-based PM tasks in nondemented older adults with the ε4 allele carriers compared with individuals without the ε4 allele carriers. In another study, Duchek et al.^25^ examined whether PM performance discriminated healthy aging from very mild dementia of the AD type, and investigated possible influences of alleles of the apolipoprotein E (APOE) gene. The AD group was impaired on the event-based PM task compared to the healthy older control groups. Moreover, PM performance was clearly affected by ε4 status in the very mild AD group, while subjects with ε4 allele carriers showed deficits in performance compared with non-carriers. The authors argued that PM may serve as a marker for early stage AD.

Jones et al.^26^ reported that PM impairments are evident in the preclinical phase of AD. In their study, 46 preclinical AD and 188 control participants were compared on PM and retrospective memory tasks 3 years before dementia diagnosis. The preclinical AD participants were impaired on both measures and the PM task where the prospective and retrospective components were similarly impaired. Interestingly, PM performance had an independent contribution to the prediction of AD over and above that of retrospective memory performance.

Using a simple event-based PM task, Kinsella et al.^27^ compared PM performance between participants with mild AD and healthy older controls. Participants were requested to remember to carry out a word substitution whenever a target word was encountered in a passage of text. The authors found that the AD group was significantly impaired compared to the healthy older adults group. Despite minimal retrospective memory demands (one target word to remember) the AD group performed close to floor-level on the task.

Martins & Damasceno^28^ recently examined prospective and retrospective memory in 20 patients with mild AD and 20 healthy controls. Results revealed that AD patients performed worse on both PM and retrospective memory tests compared with matched normal controls, but exhibited poorer performance in retrospective memory.

## Prospective memory in MCI

Recent investigations suggest that PM is impaired in individuals with MCI compared to healthy controls.

In an initial study, Kazui et al.^29^ administered the Rivermead Behavioral Memory Test to 24 patients with aMCI and compared the scores against 48 normal controls and 48 AD patients. Individuals with aMCI demonstrated significant impairment on two subtests assessing event-based PM.

Troyer and Murphy^30^ investigated both time-based and event-based PM in healthy older adults, individuals with aMCI, and AD. The aMCI group obtained lower scores on time- versus event-based tasks. However, no performance differences were observed between time- and event-based PM tasks within AD or control groups. The authors suggested that time-based PM tasks may be sensitive to the earliest cognitive changes associated with aMCI, possibly reflecting decreased self-initiation, attention switching, and/or inhibition on memory tasks due to early involvement of frontal lobe functions.

Blanco-Campal et al.^31^ used an event-based PM task, whose specificity of the instructions and perceptual salience of the PM cue, were manipulated. The results showed that the PM task was superior to two traditional RM tests for discriminating MCI of suspected AD etiology from normal controls. Conditions of non-specificity of instructions and perceptual non-salience of the PM cue proved particularly sensitive in detecting MCI, likely because they require more strategic attentional resources to monitor the PM cue.

In another study, Karantzoulis et al.^32^ used the Memory for Intentions Screening Test (MIST), a test developed specifically to assess PM performance in clinical populations.^33^ The authors found that aMCI patients performed poorly on both event- based and time-based PM tasks compared with controls. Participants in the aMCI group were also more impaired in time- than event-based PM, but the magnitude of the difference was not as large as that found by Troyer and Murphy.^30^

Schemitter-Edgecombe et al.^34^ examined the performance of healthy older adults and amnestic and nonamnestic MCI patients on multiple memory tasks including measures of PM. They administered an event-based PM task in which participants were instructed to remember a medicine bottle after a specific activity was completed. Both MCI groups performed worse than controls on the measures of PM. The impairment was not the result of forgetting the content of the PM instructions. For the MCI group, PM measures correlated significantly with executive functioning tests while the retrospective memory measure did not correlate with any of the executive functioning measures for either group. Also, the authors examined the relationship between memory deficit and everyday functional limitations, and found PM deficits to have implications for instrumental activities of daily living (IADLs), in particular, medication use and household activities.

In the study by Thompson et al.,^35^ individuals with MCI, dementia and controls played the Virtual Week game, which involves simulating the types of PM tasks that occur in everyday life (e.g., taking medication). The results indicated that both clinical groups exhibited PM difficulties compared to controls, but the magnitude of this deficit was greater among those with dementia. It has been suggested that, while other cognitive deficits contribute to these difficulties, there is something unique to prospective remembering that may be additionally disrupted in these groups.

In a recent study, Costa et al.^36^ investigated PM in subjects with aMCI, dysexecutive MCI and matched healthy controls, using an experimental procedure. Participants were instructed to perform three actions after 20 min had elapsed (time-based PM) or when the timer went off (event-based PM). Individuals with MCI had globally impaired PM, characterized by a more severe deficit of the prospective component than retrospective component, particularly on the time-based task where these deficits were attributed to disrupted executive control processes.

## Conclusion

PM is impaired in individuals with Alzheimer’s disease (AD) and mild cognitive impairment (MCI). Results from several studies have suggested that both patient groups perform significantly worse for different types of PM compared to healthy older adults.

Moreover, PM tasks which require a higher degree of self-initiated processing or executive functioning may be useful for detecting early cognitive changes in MCI. However, more studies are needed to verify the accuracy of PM tasks as an early marker of MCI and initial dementia.

Although PM is crucial for independent living and a common complaint among individuals with AD and MCI, it is not often assessed in clinical practice. The routine neuropsychological assessment of PM would help to clarify the nature and extent of PM impairment in these patients and yield valuable information about effective management and rehabilitation interventions.

## References

[1] McDaniel MA, Einstein GO, McDaniel MA, Einstein GO (2007). Prospective memory: a new research enterprise. Prospective memory: an overview and synthesis of an emerging.

[2] Chasteen A L, Park D C, Schwarz N (2001). Implementation intentions and facilitation of prospective
memory. Psychol Sci.

[3] Smith G, Della Sala S, Logie RH, Maylor EA (2000). Prospective and retrospective memory in normal ageing and
dementia: a questionnaire study. Memory.

[4] Einstein G O, McDaniel M A (1990). Normal aging and prospective memory. J Exp Psychol.

[5] Kvavilashvili L, Ellis J, Brandimonte M, Einstein GO, McDaniel MA (1996). Varieties of intention: Some distinctions and
classification. Prospective memory: theory and applications.

[6] Einstein GO, McDaniel MA, Richardson SL (1995). Aging and prospective memory: examining the influences of
self-initiated retrieval processes. J Exp Psychol.

[7] Craik FIM, Klix F, Hagendorf H (1986). A functional account of age differences in memory. Human memory and cognitive capabilities.

[8] West RL (1996). An application of prefrontal cortex function theory to cognitive
aging. Psychol Bull.

[9] McDaniel MA, Einstein GO (1992). Aging and prospective memory: Basic findings and practical
applications. Adv Learn Behav Dis.

[10] Kliegel M, McDaniel MA, Einstein G O (2000). Plan formation, retention, and execution in prospective memory: a
new approach and age-related effects. Mem Cognit.

[11] Ellis J, Brandimonte M, Einstein GO, McDaniel MA (1996). Prospective memory or the realization of delayed intentions: a
conceptual framework for research. Prospective memory: theory and applications.

[12] Shallice PW, Burgess PW (1991). Deficits in strategy application following frontal lobe damage in
man. Brain.

[13] Palmer HM, McDonald S (2000). The role of frontal and temporal lobe processes in prospective
remembering. Brain Cogn.

[14] Okuda J, Fujii T, Yamadori A (1998). Participation of the prefrontal cortices in prospective memory:
evidence from a PET study in humans. Neurosci Lett.

[15] Burgess PW, Quayle A, Frith CD (2001). Brain regions involved in prospective memory as determined by
positron emission tomography. Neuropsychologia.

[16] Burgess PW, Scott SK, Frith CD (2003). The role of the rostral frontal cortex (area 10) in prospective
memory: a lateral versus medial dissociation. Neuropsychologia.

[17] Khachaturian ZS (1985). Diagnosis of Alzheimer's disease. Arch Neurol.

[18] McKee AC, Au R, Cabral HJ (2006). Visual association pathology in preclinical Alzheimer
disease. J Neuropathol Exp Neurol.

[19] Backman L, Jones S, Berger AK, Laukka EJ, Small BJ (2005). Cognitive impairment in preclinical Alzheimer's disease: a
meta-analysis. Neuropsychology.

[20] Amieva H, Lafont S, Auriacombe S (1998). Analysis of error types in the trail making test evidences an
inhibitory deficit in dementia of Alzheimer type. J Clin Exp Neuropsychol.

[21] Huppert FA, Beardsall L (1993). Prospective memory impairment as an early indicator of
dementia. J Clin Exp Neuropsychol.

[22] Huppert FA, Johnson T, Nickson J (2000). High prevalence of prospective memory impairment in the elderly
and in early-stage dementia: findings from a population-based
study. Appl Cogn Psychol.

[23] Maylor EA, Smith G, Della Sala S, Logie RH (2002). Prospective and retrospective memory in normal aging and
dementia: an experimental study. Mem Cognit.

[24] Driscoll I, McDaniel MA, Guynn MJ (2005). Apolipoprotein E and prospective memory in normally aging
adults. Neuropsychology.

[25] Duchek JM, Balota DA, Cortese M (2006). Prospective memory and Apolipoprotein E in healthy aging and
early stage Alzheimer's disease. Neuropsychology.

[26] Jones S, Livner Å, Bäckman L (2006). Patterns of prospective and retrospective memory impairment in
preclinical Alzheimer's disease. Neuropsychology.

[27] Kinsella GJ, Ong B, Storey E, Wallace J, Hester R (2007). Elaborated spaced-retrieval and prospective memory in mild
Alzheimer's disease. Neuropsychol Rehab.

[28] Martins SP, Damasceno BP (2008). Prospective and retrospective memory in mild Alzheimer's
disease. Arq Neuropsiquiatr.

[29] Kazui H, Matsuda A, Hirono N (2005). Everyday memory impairment of patients with mild cognitive
impairment. Dem Geriatr Cogn Dis.

[30] Troyer AK, Murphy KJ (2007). Memory for intentions in amnestic mild cognitive impairment:
time- and event-based prospective memory. J Int Neuropsychol Soc.

[31] Blanco-Campal A, Coen RF, Lawlor BA, Walsh JB, Burke TE (2009). Detection of prospective memory deficits in mild cognitive
impairment of suspected Alzheimer's disease etiology using a novel
event-based prospective memory task. J Int Neuropsychol Soc.

[32] Karantzoulis S, Troyer AK, Rich JB (2009). Prospective memory in amnestic mild cognitive
impairment. J Int Neuropsychol Soc.

[33] Raskin S (2005). Memory for intentions screening test. J Int Neuropsychol Soc.

[34] Schmitter-Edgecombe M, Woo E, Greeley DR (2009). Characterizing multiple memory deficits and their relation to
everyday functioning in individuals with mild cognitive
impairment. Neuropsychology.

[35] Thompson C, Henry JD, Rendell PG, Withall A, Brodaty H (2010). Prospective memory function in mild cognitive impairment and
early dementia. J Int Neuropsychol Soc.

[36] Costa A, Perri R, Serra L (2010). Prospective memory functioning in Mild Cognitive
Impairment. Neuropsychology.

